# Case report: Sodium and chloride muscle channelopathy coexistence: A complicated phenotype and a challenging diagnosis

**DOI:** 10.3389/fneur.2022.845383

**Published:** 2022-08-23

**Authors:** Serena Pagliarani, Giovanni Meola, Melania Filareti, Giacomo Pietro Comi, Sabrina Lucchiari

**Affiliations:** ^1^Department of Neurological Sciences, Dino Ferrari Centre, IRCCS Fondazione Ca' Granda Ospedale Maggiore Policlinico, University of Milan, Milan, Italy; ^2^Department of Biomedical Sciences for Health, University of Milano, Milan, Italy; ^3^Department of Neurorehabilitation Sciences Casa di Cura del Policlinico, Milan, Italy

**Keywords:** myotonia, paramyotonia, channelopathies, *CLCN1*, *SCN4A*

## Abstract

Non-dystrophic myotonias (NDM) encompass chloride and sodium channelopathy. Mutations in *CLCN1* lead to either the autosomal dominant form or the recessive form of myotonia congenita (MC). The main symptom is stiffness worsening after rest and improving by physical exercise. Patients with recessive mutations often show muscle hypertrophy, and transient weakness mostly in their lower limbs. Mutations in *SCN4A* can lead to Hyper-, Hypo- or Normo-kalemic Periodic Paralysis or to different forms of myotonia (Paramyotonia Congenita-PMC and Sodium Channel Myotonia-SCM and severe neonatal episodic laryngospasm-SNEL). SCM often presents facial muscle stiffness, cold sensitivity, and muscle pain, whereas myotonia worsens in PMC patients with the repetition of the muscle activity and cold. Patients affected by chloride or sodium channelopathies may show similar phenotypes and symptoms, making the diagnosis more difficult to reach. Herein we present a woman in whom sodium and chloride channelopathies coexist yielding a complex phenotype with features typical of both MC and PMC. Disease onset was in the second decade with asthenia, weakness, warm up and limb stiffness, and her symptoms had been worsening through the years leading to frequent heavy retrosternal compression, tachycardia, stiffness, and symmetrical pain in her lower limbs. She presented severe lid lag myotonia, a hypertrophic appearance at four limbs and myotonic discharges at EMG. Her symptoms have been triggered by exposure to cold and her daily life was impaired. All together, clinical signs and instrumental data led to the hypothesis of PMC and to the administration of mexiletine, then replaced by acetazolamide because of gastrointestinal side effects. Analysis of *SCN4A* revealed a new variant, p.Glu1607del. Nonetheless the severity of myotonia in the lower limbs and her general stiffness led to hypothesize that the impairment of sodium channel, Nav1.4, alone could not satisfactorily explain the phenotype and a second genetic “factor” was hypothesized. *CLCN1* was targeted, and p.Met485Val was detected in homozygosity. This case highlights that proper identification of signs and symptoms by an expert neurologist is crucial to target a successful genetic diagnosis and appropriate therapy.

## Introduction

Muscle chloride and sodium channelopathy are rare non-dystrophic myotonias characterized by myotonia, a prolonged muscle contraction after brief stimuli (a brief excitation) and delayed relaxation following a voluntary contraction.

Mutations in CLC-1, encoded by *CLCN1* gene (RefSeq NC_000007.13), lead to either the autosomal dominant form (Thomsen's disease: OMIM 160800) or the recessive form (Becker's disease: OMIM 255700) of myotonia congenita (MC). Muscle chloride channel works as a homodimer, each dimer representing an ion conductance pathway, the protopore. It is requested for stabilizing the resting membrane potential, and it favors the repolarization of the membrane at the end of depolarization. An impaired channel modifies the cycle of excitability of the myocyte membrane toward hyperexcitability by slowing the return to the resting potential after depolarization. Autosomal dominant myotonia congenita is due to the presence of one dominant-negative mutation that modifies either the gating of both the protopores or the selectivity of one of the two protopores (1, 2). However, some mutations may act as dominant in some patients, and as recessive in others possibly because of incomplete penetrance (3).

The main symptom complained is stiffness worsening after rest and improving by physical exercise (warm up). Patients with recessive mutations often show muscle hypertrophy with different degree and distribution (herculean appearance), and they can suffer from transient weakness at the beginning of voluntary contraction, and this may lead to falls.

Dominant mutations in Na_v_1.4, encoded by *SCN4A* gene (NC_000017.11) can lead to Hyper-, Hypo- or Normo- kalemic Periodic Paralysis (OMIM 170500) or to different forms of myotonia [Paramyotonia Congenita (PMC): OMIM 168300; Sodium Channel Myotonia (SCM): OMIM 603967]; Severe neonatal episodic laryngospasm (SNEL): OMIM 608390, whereas recessive mutations are associated to congenital myopathy or congenital myasthenic syndromes (OMIM 614198). Sodium channel myotonia is often characterized by facial muscle stiffness, cold sensitivity, and muscle pain. The clinical symptoms are highly variable ranging from a severe neonatal presentation passing through classical SCM to mild, late-onset phenotypes (4). The warm-up phenomenon is usually present in MC patients, but it is sometimes experienced also by SCM patients. On the other hand, PMC patients experience the worsening of myotonia with the repetition of the muscle activity (paradoxical myotonia) and cold, and they could also complain about asthenia and weakness.

Often patients affected by chloride or sodium channelopathies show similar phenotypes and common clinical symptoms, making the diagnosis more difficult to reach.

Herein we present a patient affected by non-dystrophic myotonia where sodium and chloride channelopathies coexist yielding a complex phenotype with features typical of both MC and PMC. The patient's DNA harbors a previously described *CLCN1* mutation, p.Met485Val, in homozygosity, and a novel dominant *SCN4A* variation, p.Glu1607del.

It is well-established in the field of the muscle channelopathies that the application of differential EMG protocols comprehensive of exercise tests may help discriminating the causative gene (5). Nevertheless, this case highlights how crucial it is for the correct identification and attribution of clinical signs and symptoms by the expert neurologist in order to properly redirect the genetic testing and reach a correct diagnosis.

## Case description

Herein we describe a 53-year-old woman (DOB 1968) born in Sicily from parents that were first-degree cousins. The disease onset was early when she was 14-year-old, with asthenia, weakness, warm up and stiffness at her arms, and legs after physical activity mainly during volleyball. The symptoms worsened when she, with her parents, moved to the North of Italy where temperatures are cooler. At age of 45 she returned to medical attention complaining about a heavy retrosternal compression, sinus tachycardia with normal ECG, tingling and worsening of myalgia: indeed, ergometric test was interrupted for pain at lower limbs. At the age of 47, after her neuromuscular signs had worsened, she underwent an EMG test at the four limbs showing that myotonic discharges were present in all the tested muscles, especially in biceps brachii and biceps femoris bilaterally. No stimulation test according to Fournier protocol was performed. At age 49 the neurological examination showed evident lid lag and grip myotonia, both worsening with repeated contractions (paradoxical myotonia). Slight muscle weakness (MRC grade 4) at flexor neck muscles, abductors, and flexors (MRC grade 4) of her arms, and flexors (MRC grade 4) of the lower limbs was also present. She had no weakness at distal muscles, and she never complained of adynamia nor paralysis episodes. Laboratory investigations, including CK, were unremarkable. The patient referred an overall worsening of her disease through the years, with difficulties in climbing stairs, and impairing of the daily activities, her work as a hotel housekeeper being impacted significantly. Clinical features are summarized in Table 1.

**Table 1:** Proband's clinical features are recapitulated here.

**Characteristics**	
Age (yrs)	53
Age of onset (yrs)	14
*CLCN1* variant	p.Met485Val/p.Met485Val
*SCN4A* variant	p.Glu1607del/-
EMG	Myotonic discharges
Handgrip myotonia	No
Orbicularis myotonia	Yes
Lid myotonia	Severe
Paramyotonia	Yes
Muscle stiffness	Severe
Weakness	Moderate, fixed
Muscle pain	Severe
Warm up	No
Muscle hypertrophy	Severe
Triggers for myotonia	Cold

The patient's father, 75 years old, complained only for myotonic symptoms since when he had moved to the North of Italy for work reasons. As his daughter, he showed palpebral paradoxical myotonia, while he never complained about myalgia or limb stiffness. He worked as a laborer. The Patient's 68 years old mother had no complaints at all, and her neurological examination was unremarkable. Both parents, due to mild and the absence of symptoms, refused to undergo EMG, they agreed only on genetic testing.

## Diagnostic assessment and treatments

**Figure 1:**
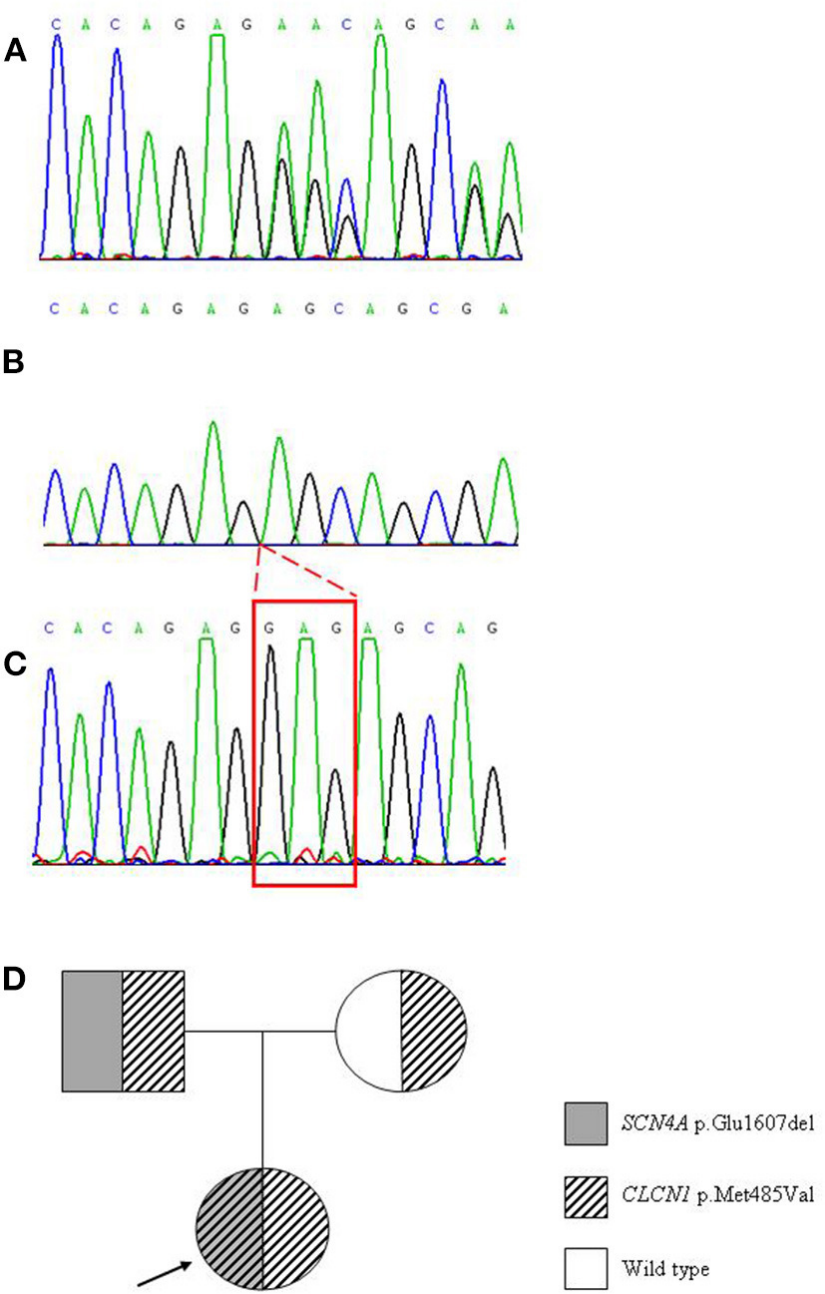
Electropherograms from Sanger sequencing showing the patient's altered pattern due to the trinucleotide deletion p.Glu1607del on *SCN4A* gene **(A)** electropherogram obtained after cloning of the PCR product carrying the mutation, in order to resolve the mutated from the wild type allele **(B)** normal pattern in a control case **(C)**. Genetic tree showing the hereditary pattern of both the mutations harbored by the proband (indicated by the arrow) **(D)**.

Instrumental examinations made at disease onset were not available. At age 46 echocardiograms detected slight atrioventricular insufficiency. Because of her difficulty in movements, she underwent lumbosacral MRI, which was negative. EMG examinations performed at age 47 showed myotonic discharges at rest in almost all tested muscles. These data together with orbicular myotonia, and the persistence of stiffness after repeated contractions led to the hypothesis of paramyotonia congenita, and to administration of mexiletine 200 mg once daily, interrupted for gastrointestinal side effects, and replaced by acetazolamide 62.5 mg three times daily without improvement of symptoms. When the patient was 48 years old, lacosamide 50 mg twice daily was tempted to obtain a better control of symptoms, but she positively responded only for a short time, after which cramps started over (Table 2). Concurrently a genetic analysis of the entire *SCN4A* gene was performed revealing a new variant, c.4819_4821delGAG harboring the p.Glu1607del (Figures 1A–C), inherited from the patient's father (Figure 1D). In consideration of the severity of myotonia especially at the lower limbs and because of the general stiffness affecting the patient, it was likely that the phenotype could not be satisfactorily explained by impairment of *SCN4A* gene alone, and the involvement of a second genetic “actor” was hypothesized. Thus, *CLCN1* gene was targeted for a further molecular analysis, and the previously reported mutation p.Met485Val was detected in homozygosity (Figure 1D). Since when the patient was 50 years old, a different pharmacological treatment has been successfully attempted based on lamotrigine 150 mg in the morning and 125 in the afternoon, obtaining improvement of both palpebral and grip myotonia, and limb stiffness.

**Table 2:** Timeline of the drug treatments administered to the patient and their effects.

**Patient's age**	**Drug**	**Dose**	**Side effects**	**Efficacy**
47	Mexiletine	200 mg sid	Gastrointestinal	Imprv. of Symptoms
47	Acetazolamide	62.5 mg tid	-	No
48	Lacosamide	50 mg bid	-	Brief imprv. of symptoms
50	Lamotrigine	150+125 mg	-	Imprv. of palpebral and grip myotonia, limb stiffness

## Discussion

This work describes a patient with non-dystrophic myotonia presenting a complex phenotype not clearly referable to the impairment of a single skeletal muscle channel. Since disease onset, the patient showed a severe range of symptoms which led neurologist to hypothesize a “stiff-person syndrome” vs. sodium channelopathy. Indeed, the presence of severe orbicular myotonia, and the absence of warm up oriented toward a sodium channelopathy. Nevertheless, the severity of myotonia especially at lower limbs and the general stiffness were not satisfactorily explained by the diagnosis of PMC based on clinical symptoms and genetic analysis of *SCN4A*, hence the involvement of a second genetic “player,” namely *CLCN1* gene, was hypothesized.

Indeed, the sequencing of the muscle chloride channel revealed a homozygous missense mutation previously described. Both parents, as expected, were heterozygous carrier. The parents did not undergo to any instrumental examination. The nucleotide change c.1453A>G (rs146457619; gnomAD 0.04%; ClinVar 280101) in exon 13 of *CLCN1* yields the missense p.Met485Val which has been reported in a number of studies with myotonia congenita, both in homozygous and compound heterozygous state, while it was detected in heterozygote state in unaffected individuals (6–11). Clinical signs related to the presence of the p.Met485Val were reported by Mazon et al. (12) in a homozygous case sharing with our patient myotonia, and weakness after intense exercise. A clinical description of this mutation in heterozygous compound with p.Ser18Thrfs^*^55 was also done by Hoche et al. (13) in a boy of German/Indian origin presenting with symptoms of severe MC, including stiffness, myotonia after rapid initiation of movements, post-myotonic weakness, muscle pain, lid, percussion, and handgrip myotonia. Functional studies by protein expression in *Xenopus oocytes* had shown that this mutation led to a severe reduction of the single channel conductance becoming strongly inwardly rectifying, compared to wild type, thus the channel was incompletely deactivated at negative voltages (7). In a recent paper by Park and MacKinnon (14) a detailed characterization by Cryo-Electron Microscope of the CLC1 structure was proposed, and the role of Met485 was depicted. This residue is placed above the external chloride binding site located into the protopore, where its flexible side chain would form a constriction near the external end of the ion pathway, and thus would modulate the chloride throughput during membrane repolarization.

The novel *SCN4A* variant c.4819_4821delGAG (p.Glu1607del) was found in heterozygous state in both the proband and her father. It falls in the final part of the transmembrane segment S6 of domain IV of Na_v_1.4 and is highly conserved among Na_v_ channels (15) and among species. This variant is not reported in gnomAD, EVS, dbSNP or ClinVar and is predicted to be dangerous by the in silico prediction tool Mutation Taster (Disease causing). The ACMG classification is uncertain significance (PM2, PM4, PP3). To date, only a bunch of in-frame deletions were found on *SCN4A*, and they were all related to sodium channelopathies. The mutation p.Glu36del was described in a patient clinically diagnosed with HypoPP and with a positive LET (long exercise test) (16); p.Lys880del was found in a Japanese patient, and was related to HyperPP (no clinical data) (17), and in a Chinese patient with PMC (no clinical data) (18). The two in-frame deletions of the C-term of Na_v_1.4 p.Glu1702del and p.Thr1700_Glu1703del were found in myotonic patients and functional studies revealed impairment of fast inactivation for both (19). Thus, although *SCN4A* related channelopathies are mostly caused by missense mutations, there is increasing evidence that little in-frame deletions may play a role. Double trouble cases carrying mutations in both sodium and chloride muscle channels are present in medical literature (20–22). The coexistence of the p.Met485Val with mutations on the *SCN4A* gene has previously been reported by Furby et al. (20) who described a young man harboring p.Gly1306Glu/p.Met485Val, affected from birth, and sharing eye lid myotonia, abundant myotonic discharges in his legs, muscle hypertrophy normalized with age, stiffness, and myalgia with the case studied herein. He had been firstly diagnosed as a case of sodium channelopathy; still genetic findings were not consistent with the type II SET (Short Exercise Test). Sequencing of *CLCN1* found a heterozygous p.Met485 Val.

Our patient was a clinical challenge for several reasons: [1] she presented an atypical course of the disease with an age of onset at 14 years, too late in comparison to a pure sodium channelopathy where symptoms are generally present since infancy. Indeed, it fits more with a chloride channelopathy where symptoms are typically late onset from the second decade; [2] the patient was misdiagnosed for many years with a diagnosis ranging from a demyelinating disorder to stiff-person syndrome to psychiatric tracts. Only the presence of myotonic discharges at EMG correctly oriented toward a skeletal muscle channelopathy. For all these reasons, also a successful pharmacological treatment was hard to reach. First, the patient underwent to mexiletine treatment 200 mg once daily which was stopped for side effects (gastrointestinal), then to acetazolamide 62,5 mg tid, stopped because was ineffective. The next treatment, lacosamide 50 mg twice daily, was administered without effect on myotonia, and later replaced by lamotrigine 150 mg in the morning and 125 mg in the afternoon eliciting positive effects on myotonia (Table 2).

The case described herein highlights that an atypical phenotype—disease onset, a mixture of symptoms and signs—should prompt not to a single channelopathy but to the coexistence of different channel impairment which could explain the complexity of the phenotype. From this perspective, the new variant on *SCN4A* gene, p.Glu1607del, appears as a novel mutation responsible for the PMC phenotype of this case.

## Patient perspective

The case described herein emphasizes that the complexity of the mixed phenotypes requires a careful clinical follow-up, and the administration of several drugs throughout the clinical course leading to an improvement in the patient's quality of life.

Written informed consent was provided by the family members involved in this study, for treatment of biological samples, genetic analysis, and sensitive data.

## Data availability statement

The dataset in this article are not readily available due to ethical and privacy restrictions. Request to access should be directed to the corresponding author.

## Ethics statement

This study was carried out in accordance with the recommendations of Fondazione IRCCS Ca' Granda Ospedale Maggiore Policlinico of Milan. All subjects gave written informed consent for genetic analysis in accordance with the Declaration of Helsinki. Written informed consent was obtained from the individual/next of kin for the publication of any potentially identifiable images or data included in this article.

## Author contributions

SP and SL contributed to the conceptualization, writing of the paper, methodology, genetic analysis, data collection, and analysis. GM and GC reviewed the paper. GM examined the patients. MF collected the blood and DNA samples. All authors reviewed and approved the paper.

## Funding

SP and GM were funded by FMM-Fondazione Malattie Miotoniche, Milano, Italy.

## Conflict of interest

The authors declare that the research was conducted in the absence of any commercial or financial relationships that could be construed as a potential conflict of interest.

## Publisher's note

All claims expressed in this article are solely those of the authors and do not necessarily represent those of their affiliated organizations, or those of the publisher, the editors and the reviewers. Any product that may be evaluated in this article, or claim that may be made by its manufacturer, is not guaranteed or endorsed by the publisher.
